# Lapis Divinus in Otorrhœa

**Published:** 1842-04

**Authors:** 

**Affiliations:** Surgeon at the London Royal Infirmary for Diseases of the Ear


*Lapis Divinus in Otorrhœa.* By Mr. Curtis, Surgeon at the London Royal Infirmary for Diseases of the Ear.—When visiting the hospitals of Prague, I found the lapis divinus in very general use in the treatment of otorrhœa, and I afterwards found it employed in a similar manner at Vienna, Munich, Berlin, Leipsic, and Hamburgh. At Prague I procured some of the lapis divinus, and the formula for its preparation; the latter is as follows:—Take sulphate of copper, nitrate of potass, and alum, of each sixteen parts—powder them separately, and then mix them well together; fuse them in a glass vessel in a sand bath, and add one part of powdered camphor; break the mass in pieces when cold. This formula is varied in different pharmacopœias, there not being quite so much of the principal ingredient, the sulphate of copper, in some, while others substitute the acetate for it; for my part I prefer the sulphate. On my return to this country I determined to give it a trial, and have experienced much advantage from its use, as the following cases will testify. I should state, however, that the preparation I employ consists of two drachms of the lapis divinus dissolved in an ounce of distilled water, a drachm of this solution being added to six ounces of rose or distilled water; this latter constituting the injection I recommend, and which I direct to be thrown into the meatus twice a day. I begin with a weak solution, increasing the strength as I find it can be borne by the patient. Thus used, it does not cause any pain; it acts as a mild stimulant and astringent, and gradually produces a cessation of the discharge.

Case 1.—Mr. M., of Dublin, aged 50, has had a discharge from the left ear for sixteen years, attended with deafness, consequent on fever; on examination with Gruber's speculum and lamp, an ulcer was detected on the membrane of the tympanum, which membrane was perforated by it. The treatment just described was adopted; he came to me for about a month, during which time he improved materially; he afterwards pursued the same plan under my directions; the ulcer healed up, the discharge ceased, and a cure was effected, his hearing being at the same time restored.

Case 2.—Mrs. F., the widow of a clergyman, deaf seven years with the left ear, attended with a profuse, offensive discharge, tinged with blood, which she attributed to her having caught cold in her confinement. It was a singular and interesting fact in this case, that the discharge seemed to alternate or be vicarious with the catamenia, diminishing when they were present, increasing on their cessation. Inspection by Gruber's lamp and speculum showed on the membranum tympani a large ulcer with rough edges, and inclined to bleed. The edges were touched with a strong solution of the lapis divinus by means of a bougie passed into the ear, the meatus being protected by the shield of the speculum, which had been previously applied with that view. Under this treatment the ulcer improved, and a cure was gradually effected by means of a weak injection. The progress of the case was somewhat retarded by erysipelatous attacks affecting the auricle: an alterative plan of treatment was adopted in this case.

Case 3.—John Poverell, aged 15, a dispensary patient, has labored under a very offensive discharge from the ear, tinged with blood, and attended with partial deafness for three years the sequelæ of scarlet fever. The membrane of the tympanum was sound. The injection was used for three weeks, at the end of which time he was cured of the discharge, and had regained his hearing.

Case 4.—Mary Lowers, aged 37, a dispensary patient, has been quite deaf for ten years with otorrhœa and polypus, resulting from a cold, according to her account. The polypus was removed with the aid of the forceps, and the neck touched with a strong solution of lapis divinus, the meatus being protected by the shield of the speculum. Under this treatment, jointly with the use of the injection, the part healed, the discharge ceased, and the hearing was restored.

